# Glassy Carbon Modified with Cationic Surfactant (GCE/CTAB) as Electrode Material for Fast and Simple Analysis of the Arsenic Drug Roxarsone

**DOI:** 10.3390/ma16010345

**Published:** 2022-12-30

**Authors:** Katarzyna Tyszczuk-Rotko, Damian Gorylewski

**Affiliations:** Faculty of Chemistry, Institute of Chemical Sciences, Maria Curie-Skłodowska University in Lublin, 20-031 Lublin, Poland

**Keywords:** roxarsone, glassy carbon, cation surfactant, square-wave adsorptive stripping voltammetry, river water

## Abstract

For the fast and simple sensing of the arsenic drug roxarsone (ROX), the development of a glassy carbon electrode (GCE) modified with cationic surfactant (cetyltrimethylammonium bromide, CTAB) material is critical. The CTAB-modified glassy carbon electrode, in contrast to the unmodified one, showed excellent behavior for electrochemical reduction of ROX using cyclic voltammetry (CV) and square-wave adsorptive stripping voltammetry (SWAdSV) techniques. CV studies reveal an irreversible reduction process of NO_2_ to NH–OH in the ROX molecule in NaAc–HAc buffer (pH = 5.6). The electrode material was characterized using CV and electrochemical impedance spectroscopy. The experiments show that the surfactant-modified material has faster electron transfer and a higher active surface area, and permits a diffusion–adsorption-controlled process. After optimization, the SWAdSV procedure with GCE/CTAB has linear ranges of 0.001–0.02 and 0.02–20 µM, and a detection limit of 0.13 nM. Furthermore, the procedure successfully determined roxarsone in river water samples.

## 1. Introduction

The arsenic drug roxarsone (ROX, 3-nitro-4-hydroxyphenyl arsenic acid) was widely used in poultry farming. It can promote animal growth, exhibits an anti-coccidian effect against the intestinal parasites and cures dysentery [1,2,3]. ROX is excreted by animals and degraded into toxic metabolites. The toxicity of ROX is increased through biotic and abiotic pathways when it is transferred into inorganic arsenic (As(III) and As(V)). Therefore, ROX can cause risks to human health and the environment. For this reason, in 1999 ROX was banned in the European Union [4]. In the U.S., it has not been officially banned by the Food and Drug Administration (FDA), but the ROX manufacturer Pfizer stopped its production in 2011 [5]. In 2019 China, one of the world’s largest producers of poultry and swine, also banned the use of ROX in animal feed [6]. However, ROX is still used in many parts of Asia, in Canada, and in South American countries [5,7]. 

Gas chromatography and high-performance liquid chromatography are the main methods used for the ROX determination [8,9]. However, these tools are costly, requiring a time- and reagent-consuming sample preparation and analysis step. Moreover, they are laborious and are not suitable for on-site analysis. Electrochemical techniques are sensitive, relatively cheap and portable, and could be alternative analysis techniques to the conventional tools. Nonetheless, there are few reports in which electrochemical techniques are applied for ROX analysis [10,11,12,13,14,15,16,17,18]. 

In the present study, we have described a fast and simple electrochemical procedure for the determination of ROX in environmental water samples using a cationic surfactant-modified glassy carbon working electrode material in conjunction with square-wave adsorptive stripping voltammetry (SWAdSV). Surfactant molecules are amphiphilic in character, with a hydrophilic group that is compatible with water on one end and a long hydrophobic tail. As regards the surfactant properties (mainly adsorption at interfaces and aggregation into supramolecular structures) in electrochemistry, many uses have been reported [19,20,21,22]. Surfactant adsorption may occur due to the electrostatic interaction, van der Waals interaction and solvation and desolvation of adsorbate and adsorbent species [22]. 

The effects of different types of surfactants, namely anionic, nonionic and cationic ones, on the reduction of ROX were examined. It was established that a cationic surfactant (cetyltrimethylammonium bromide, CTAB) adsorbed on a glassy carbon (GC) surface, which made it possible to obtain a large and active surface area and a significant increase in the reduction signal of ROX. The ROX reduction process at the CTAB-modified glassy carbon electrode (GCE/CTAB) was studied using various techniques, such as cyclic voltammetry (CV), electrochemical impedance spectroscopy (EIS) and square-wave adsorptive stripping voltammetry. 

## 2. Materials and Methods

### 2.1. Reagents and Apparatus

Ultrapurified water (>18 MW cm, Milli-Q system, Millipore, UK) was used to prepare the solutions. To prepare 10 and 1 mM solutions of roxarsone (AK Scientific, Union City, CA, USA), 96% ethanol solution (Merck, Darmstadt, Germany) was used. Sodium dodecyl sulphate (SDS), Triton X-100 and cetyltrimethylamonnium bromide (CTAB) were obtained from Merck. An acetate buffer solution (NaAc–HAc) made with CH_3_COONa and CH_3_COOH (Merck, Darmstadt, Germany) of pH 5.6 was used for analysis of ROX. For interference studies, standard solutions of Fe^3+^, Ni^2+^, Pb^2+^, Cd^2+^, Zn^2+^, Sb^3+^, Cu^2+^, NO_2_^−^, NO_3_^−^, Cl^−^ and PO_4_^3−^ (Merck, Darmstadt, Germany) were used. River water samples from the Bystrzyca River (Lublin, Poland) were spiked with appropriate concentrations of ROX and filtered using a 0.22 µm Millipore filter.

An µAutolab analyzer with GPES 4.9 and FRA 4.9 software (Eco Chemie, Utrecht, Netherlands) was applied to perform the electrochemical experiments. The CTAB-modified GCE (diameter of 1 mm), Ag/AgCl (3M KCl) and Pt wire were used as the working, reference and auxiliary electrodes, respectively. The electrode surface cleaning was performed using silicon carbide paper (SiC-paper, #2000, Buehler, Skovlunde, Denmark), alumina particle suspension (0.3 µm), a Buehler polishing pad and an ultrasonic bath (InterSonic, model IS-2, Olsztyn, Poland). 

### 2.2. Preparation of GCE/CTAB and ROX Analysis

The GCE surface was covered with CTAB during voltammetric measurements. The 0.075 M NaAc–HAc (pH = 5.6), 40 mg/L CTAB solution and a specified volume of the standard ROX solution or sample were added to 10 mL of the electrochemical cell. In the first stage, a potential of 0.5 V was applied for 10 s in order to electrochemically clean the electrode surface. Then, without applying the potential and stirring the solution, the electrode was left in the test solution for 90 s. In this step, the accumulation of CTAB and ROX onto the electrode surface takes place. Square-wave adsorptive stripping voltammograms were registered from 0 to −1.1 V with a frequency (*f*) of 350 Hz, a square-wave amplitude (*E_SW_*) of 50 mV and a step potential (Δ*E*) of 18 mV. For each voltammograms the background curve was subtracted and the baseline was corrected. 

## 3. Results and Discussion

### 3.1. GCE/CTAB Sensor Characteristics

**Figure 1:**
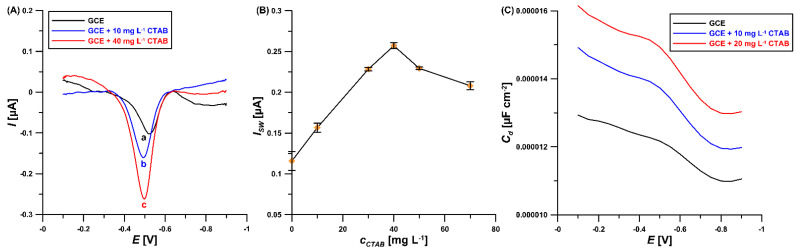
**(A**) SWV curves of 5 µM ROX in 0.05 M NaAc–HAc buffer (pH = 5.6) recorded without (a), and in the presence of 10 mg/L (b) and 40 mg/L CTAB (c). (**B**) The relationship between CTAB concentration and 5 µM ROX peak current intensity. (**C**) The differential capacity-potential curves of the double-layer interface GCE/ NaAc–HAc buffer (pH = 5.6) in the presence of 0, 10 mg/L and 20 mg/L CTAB (frequency of 200 Hz). The solution was stirred for 60 s before each measurement. The standard deviation was calculated for n = 3.

**Figure 2:**
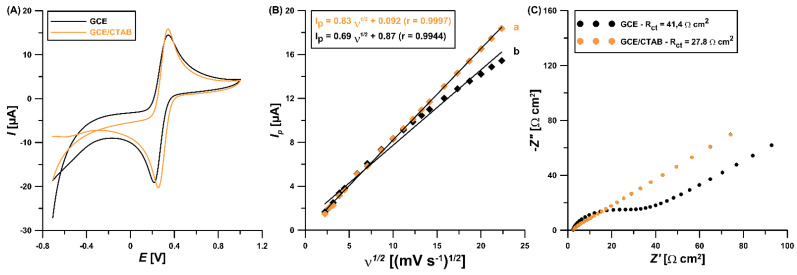
(**A**) CV curves of 5.0 mM K_3_[Fe(CN)_6_] in 0.1 M KCl at the GCE and GCE/CTAB (*υ* of 500 mV/s). (**B**) The relationship between *I_p_* and *υ*^1/2^ at the GCE/CTAB (a) and GCE (b). (**C**) Nyquist diagrams of the GCE and GCE/CTAB.

The effect of different types of surfactants, such as SDS (anionic), Triton-X 100 (non-ionic), and CTAB (cationic), on the reduction signal of ROX was studied. The peak current and peak potential of the analyte depended on the type and amount of surfactant in the supporting electrolyte. Among the studied surfactants used in the present work, 10 mg/L of SDS and Triton-X 100 caused a decrease in peak intensity (to 69.3 and 75.4% of their original values, respectively), while 10 and 40 mg/L of CTAB enhanced the ROX peak current intensity to 135.4 and 164.0% of its original value, respectively (Figure 1A). The CTAB formed a layer on the electrode surface with a high density of positive charges focusing on the exterior of the electrode surface. Therefore, molecules in anionic form are electrostatically attracted to the surface of the electrode [19,20,21,22]. According to the literature data [23] ROX has three pK_a_ values in aqueous solution (3.49, 6.38 and 9.76) and in acidic medium of pH 5.6 exists in mono-anion form. Therefore CTAB may improve, SDS may repel and Triton X-100 may hinder the ROX accumulation. Consequently, CTAB was selected for further experiments. Figure 2B demonstrates the variations in the ROX peak current intensity with the changing CTAB concentration (0–70 mg/L). The ROX analytical signal increased with the CTAB concentration up to 40 mg/L, and then the signal slowly decreased when the amount of CTAB increased. This signal increase was related to the formation of a positively charged CTAB adsorbent layer on the surface of the GCE electrode, which was confirmed using the differential capacity curves obtained without and in the presence of CTAB in the solution (Figure 1C). As can be seen, the signal of CTAB adsorption on the electrode surface (around −0.5 V) increases with increasing concentration of CTAB. 

The CTAB surface modification mechanism was supported by electrochemical impedance spectroscopic (EIS) and cyclic voltammetric (CV) studies. The CV curves of 5.0 mM K_3_[Fe(CN)_6_] as redox probe in 0.1 M KCl at the GCE and GCE/CTAB (*ν* of 500 mV/s) are presented in Figure 2B. The GCE/CTAB displays enhancement in oxidation current response (Fe^2+^ to Fe^3+^) compared to the GCE (18.4 vs. 15.4 µA, respectively). This result indicates that the active sites of the GCE increases after surface modification with surfactant. To confirm these results, the active surface areas (*A_s_*) for these electrodes were specified. The plot of *I_p_* versus ʋ^1/2^ (Figure 2B, *ν* of 5.0−500 mV/s) demonstrates linearity with an *r* value of 0.9944 for the GCE and 0.9997 for the GCE/CTAB. The *A_s_* values of these electrodes were calculated considering the slope of *I_p_* (anodic peak current) versus *ʋ*^1/2^ (square root of the *υ*) based on the Randles−Sevcik equation [24]. The *A_s_* is the maximum for the GCE/CTAB (0.728 mm^2^) in comparison with the GCE (0.601 mm^2^). Moreover, modification of the GCE surface with CTAB causes an acceleration of the electron transfer kinetics, as seen in the values of relative peak separation (*χ°*) (2.0 for the GCE and 1.4 for the GCE/CTAB, *ν* of 500 mV/s). Figure 2C presents the Nyquist plots where the diameter of the semicircle is proportional to the charge transfer resistance (*R_ct_*). From the plots, it is clear that the GCE/CTAB exhibits a smaller semicircle (*R_ct_* of 27.8 Ω cm^2^) in comparison with the GCE (*R_ct_* of 41.4 Ω cm^2^), indicating the synergistic effect of conductivity due to the CTAB-adsorbed layer promoting a rapid electron transfer in the redox probe ([Fe(CN)_6_]^3−/2−^).

### 3.2. Electrochemical Behaviour of ROX

**Figure 3:**
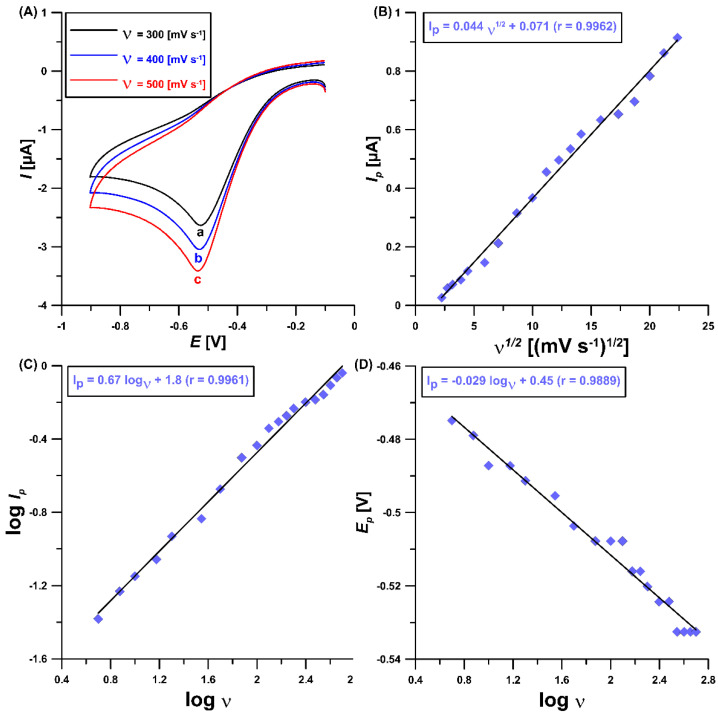
(**A)** CV curves of 5 µM ROX in 0.05 M NaAc–HAc buffer (pH = 5.6) and 40 mg/L CTAB at the GCE/CTAB. The relationship between (**B**) *I_p_* and *ʋ*^1/2^ (*υ* from 5 to 500 mV/s), (**C**) log *I_p_* and log *ʋ* and (**D**) *E_p_* and log *ʋ*.

**Figure 4:**
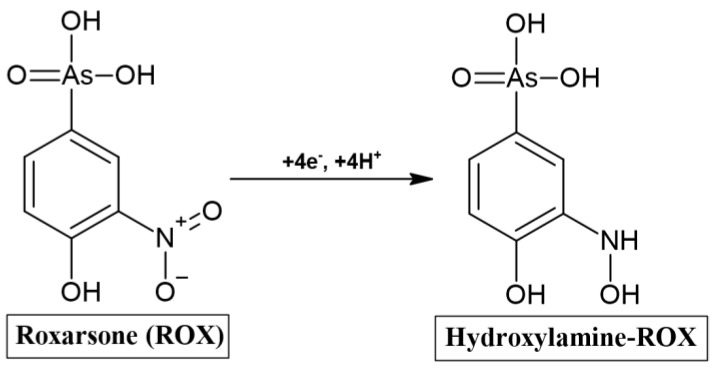
Schematic reduction mechanism of ROX at the GCE/CTAB.

Figure 3A illustrates the CV curves at the GCE/CTAB for three different scan rates (*υ* of 300, 400 and 500 mV/s) and 5 µM ROX. As can be seen, the cathodic peak associated with ROX reduction slightly shifts to more negative potentials with the υ increase. The lack of an anodic peak confirms the irreversible behavior of ROX at the GCE/CTAB. As shown in Figure 3B, a linear relationship (*r* = 0.9962) between the ROX reduction peak current (*I_p_*) and the square root of the scan rate (*ʋ*^1/2^) is observed, which suggests that the electrode reaction is a diffusion-controlled process. Moreover, in Figure 3C the relationship between log *I_p_* and log *ʋ* (r = 0.9961) is presented. The slope value of 0.67, higher than the theoretical value of 0.5, suggests a mixed, diffusion–adsorption-controlled process [25]. Additionally, the relationship between the reduction peak potential of ROX (*E_p_*) and log *ʋ* (Figure 3D) was examined. Then, using the obtained slope and Laviron’s equation [26], the number of electrons involved in the ROX electrochemical reduction process at the GCE/CTAB sensor was calculated. The received value of 4.07 suggested that four electrons were involved in the ROX reaction. According to the literature data [27,28], electrochemical reduction of nitroaryls can yield nitroso compounds, hydroxylamines and anilines as products. In each of these steps, 2 e^−^/2 H^+^ are involved. Summing up, the ROX signal at the GCE/CTAB (peak potential around −0.5 V) in NaAc–HAc buffer (pH = 5.6) comes from the irreversible reduction process of NO_2_ to NH–OH in the ROX molecule (Figure 4). 

**Figure 5:**
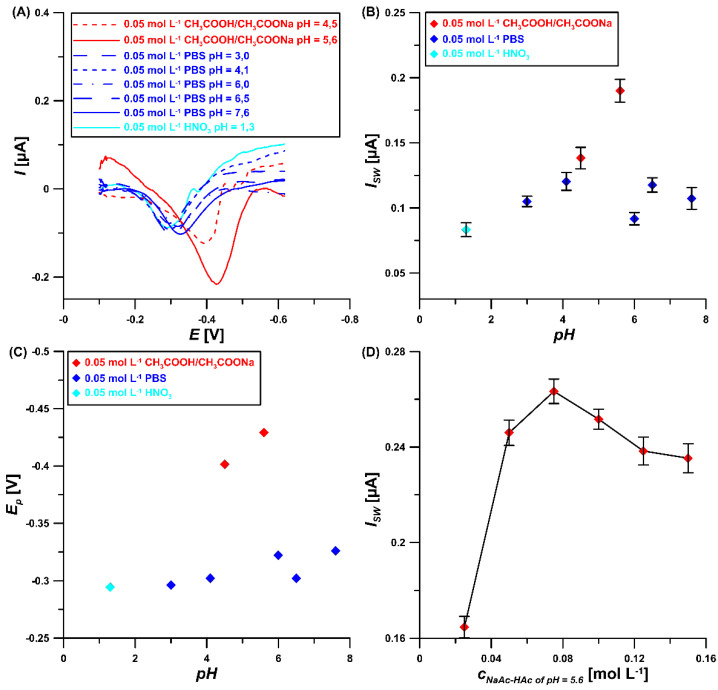
(**A**) SWV curves in different supporting electrolytes. The relationship between: (**B**) *I_SW_* and pH, (**C**) *E_p_* and pH and (**D**) *I_SW_* and the concentration of NaAc–HAc buffer of pH = 5.6. The experiments were performed for 5 µM ROX and 40 mg/L CTAB. The standard deviation was calculated for n = 3.

Moreover, the electrochemical behavior of 5 µM ROX at the GCE/CTAB at various types and pH values of the supporting electrolyte in the presence of 40 mg/L CTAB was studied (Figure 5A). As can be seen (Figure 5B,C), the peak current intensity and peak potential values depend on the pH but also on the composition of the electrolyte, which is related to its ionic strength. The potential of ROX reduction changes in different pH values, which means that protons take part in the reduction process of ROX. The highest signal with the most negative peak potential was obtained in 0.05 M NaAc–HAc buffer of pH = 5.6, so this supporting electrolyte was used in further experiments. Moreover, the effect of the NaAc–HAc buffer concentration on the 5 µM ROX signal was examined (Figure 5D). The highest signal was obtained for the 0.075 M NaAc–HAc buffer concentration. Therefore, since then this concentration value was considered as optimal.

### 3.3. Procedure Parameter Optimization Step 

**Figure 6:**
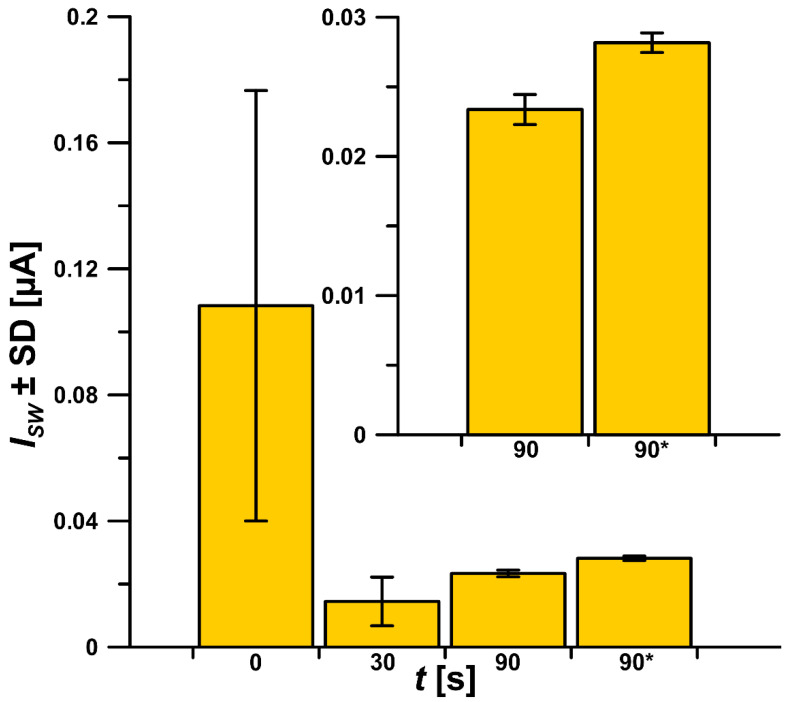
The relationship between *I_SW_* and *t* (CTAB and ROX accumulation time). ^*^ means time without stirring the solution. The standard deviation was calculated for n = 3.

The CV studies indicated that the electroreduction process of ROX at the GCE/CTAB is not purely diffusion- and adsorption-controlled. Therefore, the effect of the potential and time accumulation of ROX on its signal was tested. It was found that the use of potential values of −0.25, −0.5, 0, 0.1 and 0.25 V did not influence on the 0.05 µM ROX signal. Therefore, the accumulation of ROX was performed in open circuit potential. After electrochemical cleaning of the electrode surface (0.5 V for 10 s), the electrode was left in the stirring solution for a certain period of time, so as to adsorb the CTAB layer onto the GCE surface and accumulate ROX. As can be seen in Figure 6, the highest signal was obtained without additional time for CTAB and ROX accumulation. However, the results are subject to a huge error (RSD around 65%, n = 5). It was found that the 0.05 µM ROX signal repeatability improves with increasing time. Moreover, the application of an accumulation time of 90 s without solution stirring decreased the standard deviation of the peak current intensity. In this case the RSD was equal to 0.7% for n = 3. Further extension of the time did not increase the ROX signal or improve the repeatability.

**Figure 7:**
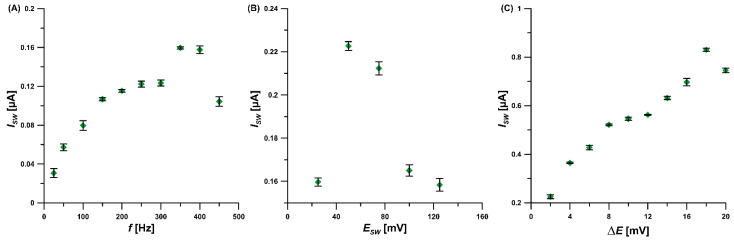
The relationship between *I_SW_* and *f* (**A**) as well as *E_SW_* (**B**) and Δ*E* (**C**) for the 1 µM ROX peak current. The standard deviation was calculated for n = 3.

Additionally, the parameters of the signal recording procedure (SWV) were optimized. First, the frequency (*f*) was changed in the range of 25–450 Hz, and the 1 µM ROX peak current intensity was measured (Figure 7A). It was found that the signal increased with *f* up to 350 Hz. This value of *f* was selected as optimal. The relationship between *I_SW_* and SWV amplitude (*E_SW_*) for the 1 µM ROX peak current was studied in the range of 25–125 mV (Figure 7B). Based on the intensity of the ROX signal, *E_SW_* of 50 mV was selected. Lastly, the relationship between *I_SW_* and step potential (Δ*E*) was tested by changing the Δ*E* value from 2 to 20 mV. The highest 1 µM ROX signal intensity was attained for the Δ*E* value of 18 mV (Figure 7C). 

### 3.4. Interference Studies

**Figure 8:**
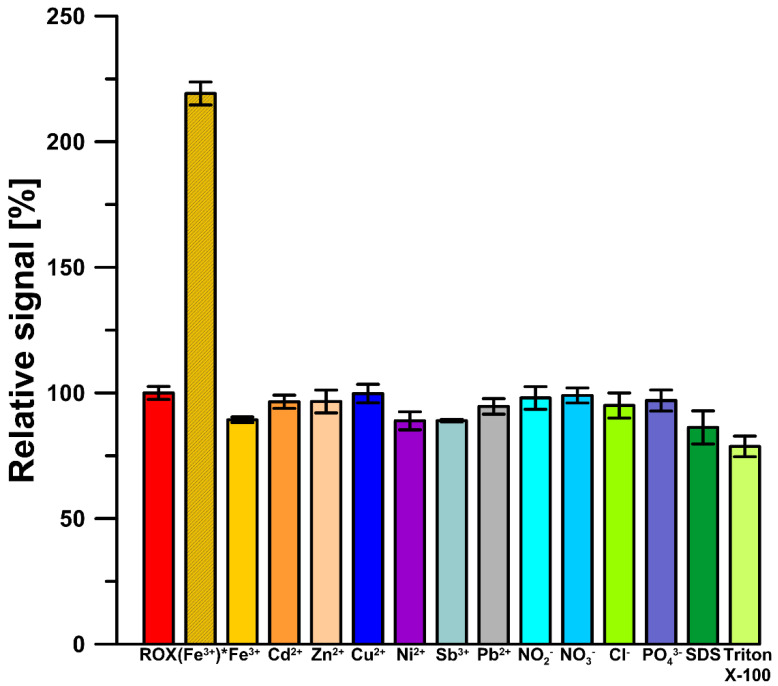
Influence of 10 µM metal ions (Fe^3+^, Ni^2+^, Pb^2+^, Cd^2+^, Zn^2+^, Sb^3+^, Cu^2+^, NO_2_^−^, NO_3_^−^, Cl^−^ and PO_4_^3−^), 10 mg/L SDS and Triton X-100 on the 1 µM ROX signal. (Fe^3+^) * without DTPA, other metal ions and surfactants in the presence of 10 µM DTPA in supporting electrolyte. The standard deviation was calculated for n = 3.

To explore the selectivity of the GCE/CTAB to ROX in environmental water samples, several interferents were added to the supporting electrolyte (0.075 M NaAc–HAc buffer of pH = 5.6 containing 40 mg/L CTAB and 1 µM ROX), and the results were investigated in percentage change in the ROX peak current intensity. The concentration of the interferents (Fe^3+^, Ni^2+^, Pb^2+^, Cd^2+^, Zn^2+^, Sb^3+^, Cu^2+^, NO_2_^−^, NO_3_^−^, Cl^−^ and PO_4_^3−^) was taken to be 10 times higher than ROX (Figure 8). As can be seen, the tested metal ions had a slight influence on the ROX signal in the presence of 10 µM DTPA. The reason for this selectivity is that the GCE/CTAB exhibited a higher adsorption capacity for ROX than for the studied metal ions and DTAP-complexed metal ions. Moreover, the effect of other surfactants (10 mg/L SDS and Triton X-100) on the ROX signal in the presence of 40 mg/L CTAB was investigated (Figure 8). The high selectivity towards ROX in this case is connected with CTAB modification of the electrode surface. CTAB counteracts surface blocking by other surfactants. 

### 3.5. SWAdSV Determination of ROX

**Figure 9:**
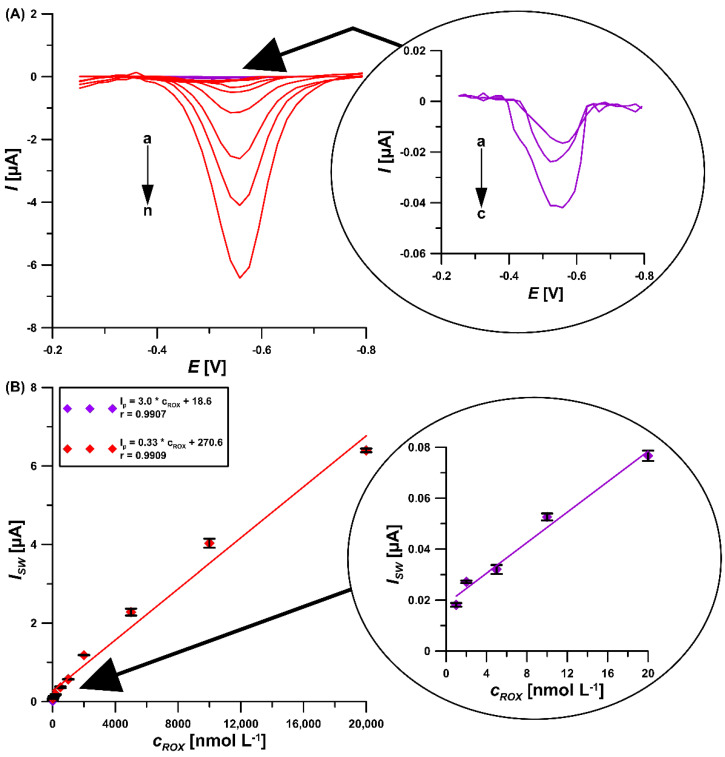
(**A**) SWAdSV curves at the GCE/CTAB in 0.075 M NaAc–HAc buffer of pH = 5.6 containing 40 mg/L CTAB and increasing concentrations of ROX (a→m, 0.001, 0.002, 0.005, 0.01, 0.02, 0.05, 0.1, 0.2, 0.5, 1, 2, 5, 10, 20 µM); (**B**) Linear calibration plots of ROX. The procedure parameters: *t* = 90 s, *f* = 350 Hz, *E_SW_* = 50 mV and Δ*E* = 18 mV. The standard deviation was calculated for n = 3.

The wide linear calibration plots at the GCE/CTAB were registered in ROX concentrations of 0.001–0.02 and 0.02–20 µM in 0.075 M NaAc–HAc buffer of pH = 5.6 containing 40 mg/L CTAB (Figure 9). The sensitivity of the sensor is 3.0 μA/nM from the linear regression equation *I_SW_* [µA] = 3.0 *c_ROX_* [nM] 18.6 and the correlation coefficient r = 0.9907. The limit of detection (LOD) and quantification (LOQ), calculated as LOD = 3SD_a_/b and LOQ = 10SD_a_/b (SD_a_—standard deviation of intercept (n = 3); b—slope of calibration plot), were 0.13 and 0.43 nM, respectively [29]. In Table 1 the SWAdSV procedure at the GCE/CTAB was compared with the other studies [8,9,10,11,12,13,14,15,16,17,28,30]. The current analytical procedure has a lower LOD compared to gas chromatography and high-performance liquid chromatography, and practically all electrochemical procedures. Only one described in paper [17] offers a comparable LOD value, on the order of 0.1 nM. Comparing all described analytical procedures, it can be concluded that the proposed SWAdSV procedure using the GCE/CTAB sensor is more economic for ROX analysis. In this case, we have a quick and simple procedure for ROX determination and preparation of the electrode itself. It is worth emphasizing the low consumption of reagents and the possibility of conducting analyses outside the laboratory.

The practicality of the SWAdSV procedure with the GCE/CTAB sensor was investigated using ROX determination of spiked river water samples (Bystrzyca River, Lublin, Poland). A defined volume of the spiked (0.02 and 2 µM of ROX) and filtered sample was directly added to the electrochemical cell. The ROX determination was carried out using the standard addition method. It is worth emphasizing that in the case of the tested samples, there was no need to introduce DTPA into the solution in order to minimize interference from metal ions. The recovery values were 107.5 and 96.7%, which confirm a satisfactory degree of accuracy of the SWAdSV procedure at the GCE/CTAB. The results reveal practical feasibility of glassy carbon modified with cationic surfactant as electrode material for analysis of the arsenic drug ROX.

## 4. Conclusions

The SWAdSV procedure with the GCE/CTAB sensor displays a highly sensitive and selective response towards the arsenic drug roxarsone (ROX) and gives wide linearity in the ranges of 0.001–0.02 and 0.02–20 µM ROX in NaAc–HAc buffer (pH = 5.6) with the sensitivity calculated as 3.0 μA/nM and a very low limit of detection of 0.00013 µM. The CTAB adsorption onto the GCE surface causes a significant influence on the increase in ROX peak current intensity (the presence of 40 mg/L of CTAB increases the signal to 164.0% of its original value), the increase in active surface area (*A_s_* of 0.601 mm^2^ for the GCE and 0.728 mm^2^ for the GCE/CTAB), the improvement of electron transfer kinetics (*χ°* of 2.0 for the GCE and 1.4 for the GCE/CTAB, ν of 500 mV/s), and the decrease in charge transfer resistance (*R_ct_* of 27.8 Ω cm^2^ for the GCE/CTAB and 41.4 Ω cm^2^ for the GCE). The proposed simple and fast procedure can be used for practical analysis of ROX in environmental water samples without the need for an initial complicated sample preparation step. 

## Figures and Tables

**Table 1:** Comparison of techniques used for ROX analysis.

Technique (Sensor)	Linear Range (µM)	LOD (µM)	Ref.
QIT-MS-PEPD	0–0.38	0.010	[8]
HPLC-HG-AFS	0.011–0.034	0.0015	[9]
DPV (WS_2_NSs/SPCE)	0.05–489.3	0.030	[10]
DPV (WS_2_ NRs/N-rGOs/SPCE)	0.1–442.6	0.075	[11]
Amperometric method (SRWO_4_ NPs/GrO/GCE)	0.035–1816.5	0.022	[12]
DPV (MCPME)	3.8–190.0	0.19	[13]
DPV (CMCPE)	0.1–1.0	0.10	[14]
Amperometric method (PrVO_4_/SPCE)	0.001–551.8	0.00004	[15]
DPV (2D-AC/GCE)	0.76–474.0	0.0015	[16]
DPV (Tm-BTC MOF/GCE) DPV (CoMn_2_O_4_-500) DPV (LaMoO)	0.00015–770 0.01–0.84 and 0.84–1130 0.025–2650	0.0001 0.002 0.0124	[17] [27] [28]
SWAdSV (GCE/CTAB)	0.001–0.02 and 0.02–20	0.00013	This work

## Data Availability

Not applicable.

## References

[1] Cortinas I., Field J.A., Kopplin M., Garbarino J.R., Gandolfi A.J., Sierra-Alvarez R. (2006). Anaerobic biotransformation of roxarsone and related N-substituted phenylarsonic acids. Environ. Sci. Technol..

[2] Stolz J.F., Perera E., Kilonzo B., Kail B., Crable B., Fisher E., Ranganathan M., Wormer L., Basu P. (2007). Biotransformation of 3-nitro-4- hydroxybenzene arsonic acid (roxarsone) and release of inorganic arsenic by Clostridium species. Environ. Sci. Technol..

[3] Sierra-Alvarez R., Cortinas I., Field J.A. (2010). Methanogenic inhibition by roxarsone (4-hydroxy-3-nitrophenylarsonic acid) and related aromatic arsenic compounds. J. Hazard. Mater..

[4] Huang L., Yao L., He Z., Zhou C., Li G., Yang B., Deng X. (2014). Roxarsone and its metabolites in chicken manure significantly enhance the uptake of as species by vegetables. Chemosphere.

[5] D’Angelo E., Zeigler G., Beck E.G., Grove J., Sikora F. (2012). Arsenic species in broiler (Gallus gallus domesticus) litter, soils, maize (*Zea mays* L.), and groundwater from litter-amended fields. Sci. Total Environ..

[6] Hu Y., Cheng H., Tao S., Schnoor J.L. (2019). China’s Ban on phenylarsonic feed additives, a major step toward reducing the human and ecosystem health risk from arsenic. Environ. Sci. Technol..

[7] Frensemeier L.M., Büter L., Vogel M., Karst U. (2017). Investigation of the oxidative transformation of roxarsone by electrochemistry coupled to hydrophilic interaction liquid chromatography mass spectrometry. JAAS.

[8] Roerdink A.R., Aldstadt J.H. (2004). Sensitive method for the determination of roxarsone using solid-phase microextraction with multi-detector gas chromatography. J. Chromatogr. A.

[9] Cui J., Xiao Y.-B., Dai L., Zhao X.-H., Wang Y. (2013). Speciation of organoarsenic species in food of animal origin using accelerated solvent extraction (ASE) with determination by HPLC-hydride generation-atomic fluorescence spectrometry (HG-AFS). Food Anal. Methods.

[10] Govindasamy M., Wang S.-F., Jothiramalingam R., Ibrahim S.N., Al-lohedan H.A. (2019). A screen-printed electrode modified with tungsten disulfide nanosheets for nanomolar detection of the arsenic drug roxarsone. Microchim. Acta.

[11] Chen T.-W., Rajaji U., Chena S.-M., Chinnapaiyan S., Ramalingam R.J. (2019). Facile synthesis of mesoporous WS2 nanorods decorated N-doped RGO network modified electrode as portable electrochemical sensing platform for sensitive detection of toxic antibiotic in biological and pharmaceutical samples. Ultrason. Sonochem..

[12] Govindasamy M., Rajaji U., Wanga S.-F., Changa Y.-J., Ramalingam R.J., Chan C.-Y. (2020). Investigation of sonochemically synthesized sphere-like metal tungstate nanocrystals decorated activated carbon sheets network and its application towards highly sensitive detection of arsenic drug in biological samples. J. Taiwan Inst. Chem. Eng..

[13] Waris M., Baig J.A., Sirajuddin, Kazi T.G., Solangi I.B., Siddiqui S., Afridi H.I. (2016). Selective Electroanalytical Method for the Determination of Roxarsone in Poultry Feed and Litter. Food Anal. Methods.

[14] Ahamad R., Barek J., Yusoff A.R., Sinaga S.M., Zima J. (2000). Determination of Roxarsone Using Carbon Paste and Amberlite LA2 Modified Carbon Paste Electrodes. Electroanalysis.

[15] Sriram B., Kogularasu S., Hsu Y.-F., Wang S.-F., Sheu J.-K. (2022). Fabrication of Praseodymium Vanadate Nanoparticles on Disposable Strip for Rapid and Real-Time Amperometric Sensing of Arsenic Drug Roxarsone. Inorg. Chem..

[16] Srivastava N.S.K., Srivastava A., Srivastava M., Prakash R. (2022). Electrochemical Sensing of Roxarsone on Natural Biomass Derived. Two-Dimensional Carbon Material as Promising Electrode Material. ACS Omega.

[17] Chinnapaiyan S., Rajaji U., Chen S.-M., Liu T.-Y., Filho J.d.O., Chang Y.-S. (2022). Fabrication of thulium metal–organic frameworks based smartphone sensor towards arsenical feed additive drug detection: Applicable in food safety analysis. Electrochim. Acta.

[18] Pereira D.F., Santana E.R., Spinelli A. (2022). Electrochemical paper-based analytical devices containing magnetite nanoparticles for the determination of vitamins B_2_ and B_6_. Microchem. J..

[19] Monnappa A.B., Manjunatha J.G., Bhatt A.S. (2020). Design of a Sensitive and Selective Voltammetric Sensor Based on a Cationic Surfactant-Modified Carbon Paste Electrode for the Determination of Alloxan. ACS Omega.

[20] Dang X., Wei Y., Hu S. (2004). Effects of Surfactants on the Electroreduction of Dioxygen at an Acetylene Black Electrode. Anal. Sci..

[21] Amrutha B.M., Manjunatha J.G., Bhatt A.S., Hareesha N. (2019). Electrochemical Analysis of Evans Blue by Surfactant Modified Carbon Nanotube Paste Electrode. J. Mater. Environ. Sci..

[22] Nayak D.S., Shetti N.P. (2016). Voltammetric Response and Determination of an Anti-Inflammatory Drug at a Cationic Surfactant-Modified Glassy Carbon Electrode. J. Surfactants Deterg..

[23] Kwon J.H., Wilson L.D., Sammynaiken R. (2014). Sorptive Uptake Studies of an Aryl-Arsenical with Iron Oxide Composites on an Activated Carbon Support. Materials.

[24] Kumari C.T.R., Mamatha G.P., Santhosh H.M. (2016). Voltammetric Detection of Trimethoprim at CTAB Modified Carbon Paste Electrode. Chem. Sci. Trans..

[25] Gosser D.K. (1993). Cyclic Voltammetry: Simulation and Analysis of Reaction Mechanism.

[26] Laviron E. (1979). General expression of the linear potential sweep voltammogram in the case of diffusion less electrochemical systems. J. Electroanal. Chem..

[27] Wirtanen T., Rodrigo E., Waldvogel S.R. (2020). Recent Advances in the Electrochemical Reduction of Substrates Involving N-O Bonds. Adv. Synth. Catal..

[28] Kokulnathan T., Rajagopal V., Wang T.-J., Huang S.-J., Ahmed F. (2021). Electrochemical Behavior of Three-Dimensional Cobalt Manganate with Flowerlike Structures for Effective Roxarsone Sensing. Inorg. Chem..

[29] Mocak J., Bond A.M., Mitchell S., Scollary G. (1997). A statistical overview of standard (IUPAC and ACS) and new procedures for determining the limits of detection and quantification: Application to voltammetric and stripping techniques. Pure Appl. Chem..

[30] Vinoth S., Govindasamy M., Wang S.F., Alothman A.A., Alshgari R.A. (2021). Surface Engineering of Roselike Lanthanum Molybdate Electrocatalyst Modified Screen-Printed Carbon Electrode for Robust and Highly Sensitive Sensing of Antibiotic Drug. Microchem. J..

